# In Vitro Evaluation of Antifungal Effects of Mineral Trioxide Aggregate and Portland Cement on *Candida Albicans*

**Published:** 2007-01-20

**Authors:** Zahed Mohammadi, Abbas Ali Khademi, Fatemeh Ezoddini Ardakani

**Affiliations:** 1 *Department of Endodontics, Dental School, Shahid Sadoughi University of Medical Sciences, Yazd, Iran*; 2 *Department of Endodontics, Dental School, Esfahan University of Medical Sciences, Esfahan, Iran*; 3 *Department of Oral and Maxillofacial Radiology, Dental School, Shahid Sadoughi University of Medical Sciences, Yazd, Iran*

**Keywords:** *Candida Albicans*, Mineral Trioxide Aggregate, Portland Cement

## Abstract

**INTRODUCTION:** *Candida Albicans* (CA) is by far the most common yeast of oral infections, including endodontic infections. The aim of this study was to evaluate and compare the antifungal effect of white-colored mineral trioxide aggregate (WMTA) and Portland Cement (PC) using a tube-dilution test.

**MATERIALS AND METHODS:** WMTA and PC were tested freshly mixed and after 24 h. The experiment was performed in 24-well culture plates. Fifty wells were used and divided into four experimental groups (freshly-mixed WMTA freshly-mixed PC, 24 h-set WMTA, and 24 h-set PC) of 10 wells each and control groups of five wells each. Plates of Sabouraud dextrose agar mixed with CA served as positive control and Sabouraud dextrose agar without CA served as negative control. Fresh inoculate of CA was prepared by growing an overnight culture from a stock culture. Aliquots of CA were then taken from the stock culture and plated on the agar compound of the experimental and control groups. All plates were incubated at 37°C for1h, 24 h, and 72 h. Growth of fungi was monitored daily by the presence of turbidity. Kruskal-Wallis test was used for data analysis.

**RESULTS:** Findings showed that in the freshly mixed as well as 24 h-set WMTA and PC, fungal growth was observed during 1 h incubation; whereas by increasing the incubation time, no fungal growth was observed in 24 h and 72 h.

**CONCLUSION:** It was concluded that WMTA and PC (freshly mixed and 24-h set) were effective against CA.

## INTRODUCTION

Microorganisms play an essential role in pulpal and periapical diseases (1-3). Throughout the past decades, it has been well known that yeasts can be isolated from infected root canals (4-5). The occurrence of yeasts reported in infected root canals varies from 1-17 percent (6).

It has been shown that fungi colonization resulting in radicular pathosis can be associated with failing root canal treatments (7-11). The most commonly recovered fungi were *Candida Albicans* (CA) (7,11,12). CA also showed the ability of colonization root canal walls and penetration into dentinal tubules (13).

Factors affecting the colonization of the root canal by fungi are not fully understood. However, it seems that some predisposing factors of this process are certain intracanal medicaments, local and systemic antibiotics, and previous unsuccessful endodontic treatment (14). It has been hypothesized that the reduction of specific bacteria in the root canal during endodontic treatment may allow fungi overgrowth in low nutrition environment (6). Further, fungi such as CA may gain access to the root canal because of coronal leakage (6).

Failures of initial endodontic treatment can often be successfully recovered by orthograde retreatment or endodontic surgery. Elimination of the microbial flora and infected tissue, as well as complete seal of the root canal system, to prevent future recontamination will enhance treatment success (15). MTA has become a popular material to seal off communications between the root canal system and external surface of the root (15). It has been mostly used as a retrograde filling material and as a sealant of root perforations (16).

MTA is marketed in gray-colored and white-colored preparations; both are 75% PC, 20% bismuth oxide, and 5% gypsum by weight (17). In recent years, the use of the white-colored preparation became more popular.

MTA is a powder consists of fine hydrophilic particles that form a colloidal gel in the presence of water or moisture, and solidifies to form hard cement within approximately 3h (16). The main components of the gray-colored formula are tricalcium oxide, tricalcium silicate, bismuth oxide, dicalcium, silicate, tricalcium aluminate, tetracalcium alumino-ferrite, and calcium sulfate dehydrate (17). The WMTA lacks tetracalcium aluminoferrite (17).

PC contains the same chemical elements as MTA except for bismuth oxide in MTA (18). Further, MTA and PC are almost macroscopically and microscopically identical when analysed by X-ray analysis (19). To date, studies comparing the antifungal effects of MTA to PC are very limited (20-21). These studies evaluated the antifungal effects of MTA, PC and some other materials using the agar diffusion method. Estrela *et al.* found that there was no statistical difference between the antifungal activity of MTA and PC against CA (20). In another study Sipert *et al.* evaluated the antimicrobial activity of MTA, PC and three root canal sealers (21). Their findings showed that both MTA and PC exhibited antifungal activity.

The purpose of this study was to compare the antifungal effects of WMTA and PC against *Candida Albicans* in three exposure periods using the dilution-tube-susceptibility test.

## MATERIALS AND METHODS

The antifungal activity of WMTA (ProRoot MTA, Dentsply, Tulsa, OK, USA) as well as PC (type II) against CA was evaluated (freshly-mixed and 24h-set). Stock cultures of clinically isolated CA provided by the Microbiology Laboratory of Sadoughi University (Yazd, Iran) were maintained in Sabouraud agar plate.

A suspension was prepared by transferring three colonies from the Sabouraud agar plate using a sterile 4mm diameter platinum loop to 10 mm of Sabouraud infusion broth in a sterilized 10 mm screw-capped test tube and then incubated at 37°C for 1 week.

Two test tubes were prepared. The experiment was performed in plastic tissue-culture clusters containing 24 wells each with an inner diameter of 16 mm. A total of fifty wells were used and divided into four experimental groups (freshly-mixed WMTA , freshly-mixed PC, 24 h-set WMTA, and24 h-set PC) of 10 wells each and control groups of five wells each.

In the experimental group 1, 1 g of WMTA was mixed at the bottom of each culture well. In the experimental group 2, 1 g of PC was mixed at the bottom of each culture well. In groups 3 and 4, 24 h-set WMTA, and PC were used, respectively. For the positive control group, 1 ml of Sabouraud infusion-broth media was mixed with 1 ml of CA suspension in each culture well. In the negative control group, 2 ml of Sabouraud broth infusion was placed in each culture well. The culture plates of all experimental and control groups were then incubated anaerobically at 37^°^C and evaluated at 1, 24 and 72 h time periods. At each time period, aliquots of 0.1 ml were taken from each well and transferred to tubes containing 5 mL of fresh Sabouraud infusion broth. All tubes were incubated at 37^°^C and monitored for the consecutive 7 days.

Growth of the fungi was monitored daily by the presence of turbidity in the tubes. Turbidity was monitored with unarmed eye.

The results were analyzed statistically using Kruskal-Wallis test at 95% level of confidence.

## RESULTS

The negative control showed no fungal growth in all experimental periods, whereas the positive control demonstrated entirely fungal growth, which confirms the method.

Evaluation of the freshly mixed groups demonstrated fungal growth during 1-h incubation of CA with WMTA as well as PC. However, by increasing the incubation time, there was no growth in 24 h and 72 h. The same results were obtained in 24-h set groups. Statistically, there was no significant difference between the freshly-mixed and 24h-set MTA (P>0.05) nor between WMTA and PC (P>0.05) in all observational periods. All cell culture wells had the same results in each experimental group.

## DISCUSSION

The method used in the present study is the dilution-tube susceptibility test, which is an effective method for evaluating the antifungal and antibacterial properties of any filling material or solution (22). This method allows direct contact between fungal cells and the material. Sabouraud agar is a commonly used medium for the isolation of oral yeasts. The pH of the medium is quite acidic (usually 5.6) allowing the growth of yeasts and aciduric organisms, whereas most bacteria are inhibited (6). CA frequently associated with failing root canal treatments, has the ability to form biofilm on different surfaces (7). This property is one of the reasons why this species is considered to be more pathogenic than species that are less able to form biofilm (7).

Considering the physical, chemical, and, biological similarities, it is important to compare the antifungal effect of MTA and PC.

The results of the present study showed that freshly mixed and 24-h set MTA and PC is effective against CA in 24 and 72h.

Al-Nazhan and Al-Judai (22) showed that WMTA to be effective in killing CA in vitro for a period of up to 72h.

On the other hand, Estrela *et al.* (20) found that the antifungal activity of Gray-colored MTA against CA was limited during a 48h.

In another in vitro study, Sipert *et al.* (21) evaluated the antifungal activity of MTA, PC, and three root canal sealers (Sealapex, Fill Canal, and EndoRez). They found that antifungal effect of MTA was not significantly different from PC, but was significantly less than Sealapex, Fill Canal. Furthermore, EndoRez showed no antifungal activity. Both abovementioned studies used the agar diffusion method. However, it seems that the dilution-tube susceptibility test to be a more accurate procedure (22). To date, there is no evidence of the antifungal activity of MTA against CA for periods longer than 3 days. However, it has been shown that MTA has a soluble fraction mainly composed of calcium hydroxide and the water in contact with MTA had a high alkaline PH ranging of 11.94 to 11.99 (23). A long-term study demonstrated that MTA did maintain a high pH for 78 days (24). It is therefore possible that MTA and calcium hydroxide possess similar antimicrobial action. In this regards, several studies evaluated the susceptibility of CA to calcium hydroxide. Waltimo *et al.* (25) studied the susceptibility of common oral Candida species to saturated aqueous calcium hydroxide solution. They found that the sensitivity of the CA strains was relatively low for short-term exposure, however, after 6-h incubation, 99.9% of CA strains were killed. On the other hand, Barbosa *et al.* (26) found that a saturated solution of calcium hydroxide was effective in killing CA already after 3 min of incubation. Siqueira *et al.* (27) evaluated the antifungal activity of different intracanal medicaments against CA and found that calcium hydroxide completely eliminated the fungus from bovine dentine after 7 days. They also found that a combination of calcium hydroxide and paramonochlorophenol eliminated CA within 1h. Estrela *et al.* (20) reported that an aqueous preparation of calcium hydroxide paste in saline was more effective in inhibiting CA growth than MTA or PC. Al-Hezaimi *et al.* (15) evaluated the antifungal activity of different concentrations of WMTA on CA *in vitro* in various exposure periods. Their findings showed a direct correlation between MTA concentration and its inhibition effect on the growth of CA. MTA in concentration of 50mg/ml inhibited the growth of CA in any of the time periods tested (1, 24, 48, 72h). In concentration of 25mg/ml, MTA inhibited the growth of CA at 1 and 24h.

In lower concentrations, MTA did not inhibit the growth of CA. Mohammadi *et al.* (28) evaluated the effect of WMTA and GMTA against CA in three exposure periods (1, 24, 72h). The results showed that in the freshly mixed as well as 24 h-set MTA cements, fungal growth was observed during 1-h incubation, whereas by increasing the incubation time, no fungal growth was observed in 24 and 72 h. Extrapolation of the results of this *in vitro* study to clinical situations must be done with caution. Sealing ability and biocompatibility of MTA are more important from the clinical point of view. It is suggested that antifungal activity of MTA and PC be investigated for longer periods of time.

## CONCLUSION

In conclusion, within the limits of the present *in vitro* study, WMTA as well as PC exert an antifungal action against CA for periods of up to 3 days.

## References

[1] Kakehashi S, Stanley HR, Fitzgerald RJ (1965). The effects of surgical exposure of dental pulps in germ-free and conventional laboratory rats. Oral Surg Oral Med Oral Pathol.

[2] Moller AJ, Fabricius L, Dahlen G, Ohman AE, Heyden G (1981). Influence on periapical tissues of indigenous oral bacteria and necrotic pulp tissue in monkeys. Scand J Dent Res.

[3] Sundqvist G (1992). Ecology of the root canal flora. J Endod.

[4] Molander A, Reit C, Dahlen G, Kvist T (1998). Microbiological status of root-filled teeth with apical periodontitis. Int Endod J.

[5] Sen BH, Safavi KE, Spangberg LS (1995). Observation of bacteria and fungi in infected root canals and dentinal tubules by SEM. Endod Dent Traumatol.

[6] Waltimo TMT, Haapasalo M, Zehnder M, Meyer J (2004). Clinical aspects related to endodontic yeast infections. Endod Topics.

[7] Siqueira JF Jr, Sen BH (2004). Fungi in endodontic infection. Oral Surg Oral Med Oral Pathol.

[8] Molander A, Reit C, Dahlen G, Kvist T (1998). Microbiological state of root filled teeth with apical periodotitis. Int Endod J.

[9] Nair PNR, Sjogren U, Krey G, Kahnberg KE, Sundqvist G (1990). Intraradicular bacteria and fungi in root filled asymptomatic human teeth with therapy resistant periapical lesions: a long-term light and electron microscopic follow-up study. J Endod.

[10] Peciuliene V, Reynaud AH, Balciuniene I, Haapasalo M (2001). Isolation of Yeasts and entire bacteria in root-filled teeth with apical periodontitis. Int Endod J.

[11] Waltimo TMT, Siren EK, Torkko HL, Olsen I, Haapasalo M (1997). Fungi in therapy-resistant apical periodontitis. Int Endod J.

[12] Baumgartner JC, Watts CM, Xia T (2000). Occurrence of Candida albicans in infection of endodontic origin. J Endod.

[13] Sen BH, Safavi KE, Spangberg LS (1997). Growth patterns of Candida albicans in relation to radicular dentin. Oral Surg Oral Med Oral Pathol.

[14] Pinheiro ET, Gomes BP, Ferraz CC, Souza EL, Teixeira FB, Souza-Filho FJ (2003). Microorganisms from canals of root-filled teeth with apical periodontitis. Int Endod J.

[15] Al-Hezaimi K, Al-Hamdan K, Naghshbandi J, Oglesby S, Simon JHS, Rotstein I (2005). Effect of white-colored mineral trioxide albicans in different concentrations on Candida albicans in vitro. J Endod.

[16] Torabinejad M, Chivian N (1999). Clinical applications of mineral trioxide aggregate. J Endod.

[17] Asgary S, Parirokh M, Eghbal MJ, Brink F (2005). Chemical differences between white and gray mineral trioxide aggregate. J Endod.

[18] Asgary S, Parirokh M, Eghbal MJ, Brink F (2004). A comparative study of white mineral trioxide aggregate and white Portland cements using X-ray microanalysis. Aust Endod J.

[19] Wucherpfennig Al, Green DB (1999). Mineral trioxide aggregate vs. Portland cement: two biocompatible filling materials. J Endod.

[20] Estrella C, Bammann LL, Estrella CRA, Silva RS, Pecora JD (2000). Antimicrobial and chemical study of MTA, Portland cement, calcium hydroxide paste, sealapex, and Dycal. Braz Dent J.

[21] Sipert CR, Hussne RP, Nishiyama CK, Torres SA (2005). In vitro antimicrobial activity of Fill Canal, Sealapex, Mineral trioxide aggregate, Portland cement and EndoRez. Int Endod J.

[22] Al-Nazhan S, Al-Judai A (2003). Evaluation of antifungal activity of mineral trioxide aggregate. J Endod.

[23] Fridland M, Rosado R (2003). Mineral trioxide aggregate solubility and porosity with different water-to-powder rations. J Endod.

[24] Fridland M, Rosado R: MTA solubility (2005). A long-term study. J Endod.

[25] Waltimo TMT, Siren EK, Orstavik D, Haapasalo M (1999). Susceptibility of oral Candida species to calcium hydroxide in vitro. Int Endod J.

[26] Barbosa SV, Spangberg LSW, Almeida D (1994). Low surface tension calcium hydroxide solution is an effective antiseptic. Int Endod J.

[27] Siqueira JF Jr, Rocas IN, Lopes HP, Magalhaes FAC, Uzeda M (2003). Elimination of Candida albicans infection of the radicular dentin by intracanal medications. J Endod.

[28] Mohammadi Z, Modaresi J, Yazdizadeh M (2006). Evaluation of the antifungal effects of mineral trioxide aggregate materials. Aust Endod J.

